# Neuroprotective Effects of 7-Geranyloxycinnamic Acid from *Melicope lunu ankenda* Leaves

**DOI:** 10.3390/molecules25163724

**Published:** 2020-08-15

**Authors:** Zeinab Abdulwanis Mohamed, Enas Mohamed Eliaser, Mohammed Sani Jaafaru, Norshariza Nordin, Costas Ioannides, Ahmad Faizal Abdull Razis

**Affiliations:** 1 Natural Medicines and Products Research Laboratory, Institute of Bioscience, Universiti Putra Malaysia, UPM Serdang 43400, Selangor, Malaysia; zeinab9008@gmail.com (Z.A.M.); enaseliaser@gmail.com (E.M.E.); 2 Department of Biology, Faculty of Science, El-Mergib University, El Khums, Libya; 3 Department of Biochemistry, Kaduna State University, Main Campus, Kaduna PMB 2339, Nigeria; biojafar@gmail.com; 4 Department of Biomedical Sciences, Faculty of Medicine and Health Sciences, Universiti Putra Malaysia, UPM Serdang 43400, Selangor, Malaysia; shariza@upm.edu.my; 5 Faculty of Health and Medical Sciences, University of Surrey, Guildford, Surrey GU2 7XH, UK; c.ioannides@surrey.ac.uk; 6 Laboratory of Food Safety and Food Integrity, Institute of Tropical Agriculture and Food Security, Universiti Putra Malaysia, UPM Serdang 43400, Selangor, Malaysia; 7 Department of Food Science, Faculty of Food Science and Technology, Universiti Putra Malaysia, UPM Serdang 43400, Selangor, Malaysia

**Keywords:** *Melicope lunu-ankenda*, 7-geranyloxycinnamic acid, neuroprotection, anti-apoptosis, oxidative stress

## Abstract

Neurodegenerative diseases (NDDs) are chronic conditions that have drawn robust interest from the scientific community. Phytotherapeutic agents are becoming an important source of chemicals for the treatment and management of NDDs. Various secondary metabolites have been isolated from *Melicope lunu-ankenda* plant leaves, including phenolic acid derivatives. However, their neuroprotective activity remains unclear. Thus, the aim of this study is to elucidate the in vitro neuroprotective activity of 7-geranyloxycinnamic acid isolated from *Melicope lunu-ankenda* leaves. The neuroprotective activity was evaluated in differentiated human neuroblastoma (SH-SY5Y) cells by monitoring cell viability using 3-(4,5-dimethylthiazol-2-yl)-2,5-diphenyltetrazolium bromide (MTT). Moreover, the potential to impair apoptosis in differentiated cells was investigated employing the Annexin V-FITC assay, acridine orange and propidium iodide (AO/PI) staining, and fluorescence microscopy. Morphological assessment and ultrastructural analysis were performed using scanning and transmission electron microscopy to evaluate the effect of 7-geranyloxycinnamic acid on surface morphology and internal features of the differentiated cells. Pre-treatment of neuronal cells with 7-geranyloxycinnamic acid significantly protected the differentiated SH-SY5Y cells against H_2_O_2_-induced apoptosis. Cytoskeleton and cytoplasmic inclusion were similarly protected by the 7-geranyloxycinnamic acid treatment. The present findings demonstrate the neuroprotective potential of 7-geranyloxycinnamic acid against H_2_O_2_-induced neurotoxicity in neuronal cells, which is an established hallmark of neuronal disorders.

## 1. Introduction

Neurodegenerative diseases including Alzheimer’s disease (AD), Huntington’s disease (HD), Parkinson’s disease (PD) and amyotrophic lateral sclerosis (ALS) are distinguished by the slow loss of neuronal function [1,2], and their pathogenesis has been linked to oxidative stress, a condition induced by excessive production of reactive oxygen species (ROS) including hydrogen peroxide (H_2_O_2_) [3,4]. H_2_O_2_ is produced during defense against pathogens, aerobic respiration and cellular metabolism [5], but uncontrolled elevated intracellular levels of H_2_O_2_ damage proteins and lipids, and induce apoptosis or necrosis [6,7]. The brain is highly vulnerable to excessive oxidation due to its lipid-rich content, high oxygen demand and low antioxidant capacity [8]. Mitochondrial dysfunction frequently involves aberrant H_2_O_2_ generation, which is associated with the pathogenesis of neurodegenerative diseases. Increased oxidative stress elevation is seen in the brain of patients with neuronal disorders [9,10]. In most neurodegenerative diseases, excessive generation of H_2_O_2_ or a loss of function of the antioxidant system gives rise to oxidative injury of biological molecules, resulting in the initiation of the processes that lead to cell death. Therefore, overproduction of H_2_O_2,_ even to a small extent, requires increased antioxidant activity to afford protection [11,12].

**Figure 1:**
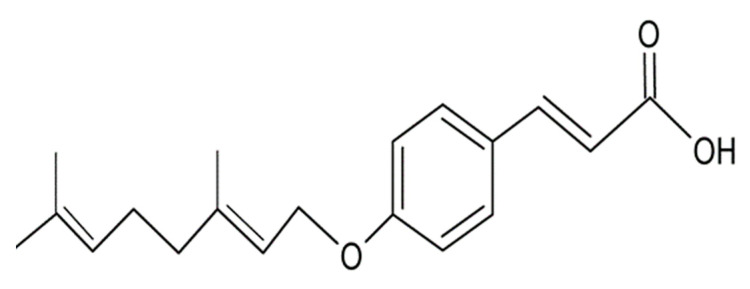
Chemical structure of 7-geranyloxycinnamic acid.

Phytochemicals have been used to alleviate and treat symptoms of neuronal disorders; some of these exert antioxidant and anti-inflammatory effects [13,14]. A number of secondary metabolites have been isolated from *Melicope lunu-ankenda* (*Rutaceae*), known locally in Malaysia as “tenggek burung” [15,16], such as polyphenols including hydroxycinnamic acid derivatives and hydroxybenzoic acid derivatives [17]. Polyphenols, including phenolic acids, have been demonstrated to exert neuroprotective activity through the prevention of protein oxidation, ROS generation and lipid peroxidation as well as anti-apoptotic and anti-inflammatory activities [18,19,20]. Hydroxycinnamic acid derivatives are widely distributed in plants and have been shown to possess significant antioxidant and anti-inflammatory activities, and to afford protection of neuronal cells [21]. These pharmacological properties correlated with the treatment of neurological disorders by impairing oxidative stress and inflammation, the two hallmarks of neurodegenerative diseases (NDDs) [22]. Studies conducted in vitro indicated that hydroxycinnamic acid derivatives exhibit antioxidant activity against reactive oxygen species and provide protection to neurons against H_2_O_2_-induced oxidative stress [23]. 7-geranyloxycinnamic acid (Figure 1) is a hydroxycinnamic acid derivative isolated from *Melicope lunu-ankenda* leaves (*Rutaceae*) [24]. The neuroprotective activity of 7-geranyloxycinnamic acid against H_2_O_2_-induced toxicity was assessed in the SH-SY5Y neuroblastoma cells line. In addition, the effect of this hydroxycinnamic acid derivative on internal morphological features (cytoplasmic inclusion) and surface ultrastructural features (topography and composition of cells surface) were also investigated in order to elucidate potential mechanisms of action. 

## 2. Results and Discussion

### 2.1. Complete Differentiation of SH-SY5Y Cells into Neurons

**Figure 2:**
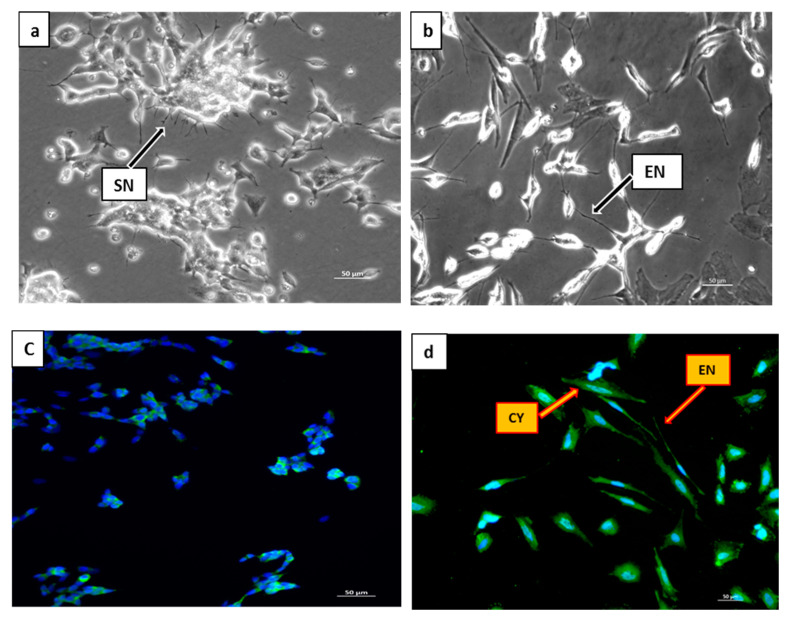

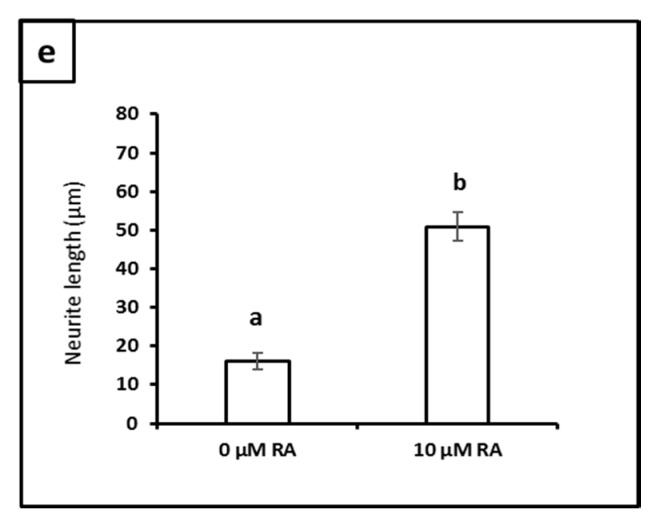
Fluorescence microscopy to confirm the differentiation process of human neuroblastoma (SH-SY5Y) cells. (**a**) Phase contrast of undifferentiated cells maintained in complete growth media. (**b**) Phase contrast of differentiated cells cultured in complete growth media treated with 10 µM of retinoic acid (RA). (**c**) Fluorescence micrograph of undifferentiated cells cultured in complete growth media and stained with 4′,6-Diamidine-2′-phenylindole dihydrochloride (DAPI). (**d**) Fluorescence micrograph of differentiated cells cultured in complete growth media treated with 10 µM of RA and stained with DAPI, increases in green fluorescent intensity indicated highly expressed tuj-1 in both cytoplasm and neurites. CY = cytoplasm, EN = extended neurites, SN = short neurites. Magnification (×20). (**e**) Neurite length in undifferentiated (0 µM RA-treated) and differentiated (10 µM RA-treated) SH-SY5Y cells after 7 days of differentiation. Data are displayed as means ± SD of triplicate experiments; means with different letters denote significant difference (*p* < 0.05).

To confirm the differentiation of SH-SY5Y cells to full neurons with retinoic acid (RA), the cell morphology was analyzed (Figure 2a–e). The effects of 10 µM RA on SH-SY5Y cell morphology were well characterized and extended neurites were observed as early as 24 h after treatment. Differentiated cells showed extended neurites as compared to undifferentiated cells having short neurites (Figure 2a). After 7 days of treatment with retinoic acid, extensive neurite outgrowth of the cells was observed (Figure 2b). Neurite length of differentiated SH-SY5Y cells increased markedly as compared to undifferentiated cells (Figure 2e). Therefore, 7-day-RA-differentiated cells were used as an experimental model [25]. Indeed, one of the most common methods to differentiate SH-SY5Y cells is through using retinoic acid at its physiological concentration (10 µM) [25,26,27,28]. The human neuroblastoma (SH-SY5Y) cells line has recently been used as a dopaminergic neuronal model for neuronal disorders such as Parkinson’s disease (PD), Alzheimer’s disease (AD), Huntington’s disease (HD), multiple sclerosis (MS) and Amyotrophic lateral sclerosis (ALS) [25,26,27,28]. SH-SY5Y neuronal cells possess several characteristics that render them a useful model of dopaminergic neurons, such as the ability to synthesize dopamine (DA), express dopamine transporters (DAT), which are known as DA homeostasis regulators, and they can also be differentiated into neuronal phenotype by using differentiation inducers such as retinoic acid [29,30].

### 2.2. Confirmation of the Differentiation Process by Immunocytochemistry Assay

The differentiation of SH-SY5Y cells by all *trans* retinoic acid (RA) was ascertained immunochemically (ICC) using phase contrast microscopy. Undifferentiated and 7-day retinoic acid (RA) differentiated cells were analyzed to compare the intensity of fluorescence of the class III β-tubulin (tuj-1) expression. Following differentiation of the SH-SY5Y cells by RA, the class III β-tubulin (tuj-1) neuronal marker is expressed [28]. In this study, weak green fluorescence was observed in a small number of undifferentiated cells (control) (Figure 2c). The intensity of green fluorescence in the cytoplasm and neurites of differentiated cells increased significantly (Figure 2d), indicating that class III β-tubulin (tuj-1) was highly expressed, thus confirming the differentiation process of SH-SY5Y cells. Incubation of SH-SY5Y cells with 10 µM RA may alter neuronal properties including but not limited to extension of neurons and the expression of certain neuron-specific markers [31,32]. 

### 2.3. Effect of 7-Geranyloxycinnamic Acid on SH-SY5Y Cell Viability and H_2_O_2_-Induced Cytotoxicity

**Figure 3:**
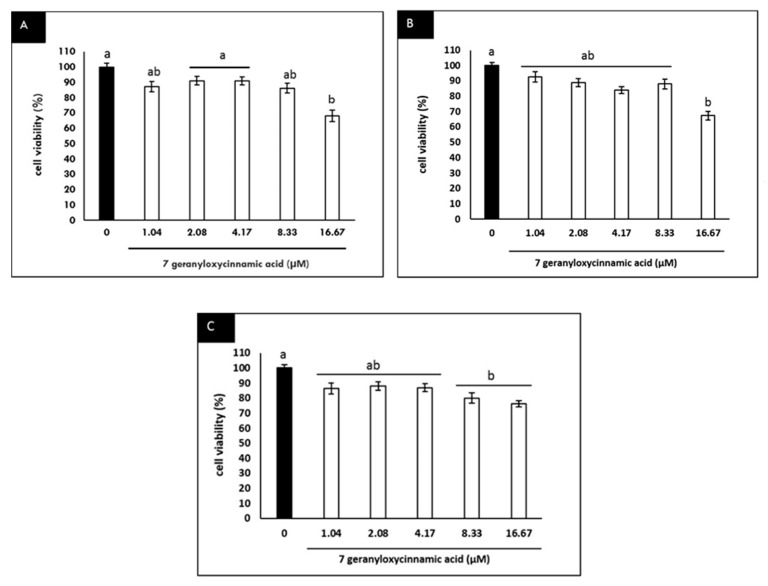
Cytotoxicity of 7-geranyloxycinnamic acid in differentiated SH-SY5Y cells at various concentrations (1.04–16.67 µM). (**A**) 24 h incubation; (**B**) 48 h incubation; (**C**) 72 h incubation. Data are displayed as means ± SD of triplicate experiments with different letters denoting significant difference (*p* < 0.05).

At the concentrations of 7-geranyloxycinnamic acid used in this study (1.04–16.67 µM), the treated cells remained viable, except those treated with the highest concentration, where a lower cell viability was evident (Figure 3A–C).

**Figure 4:**
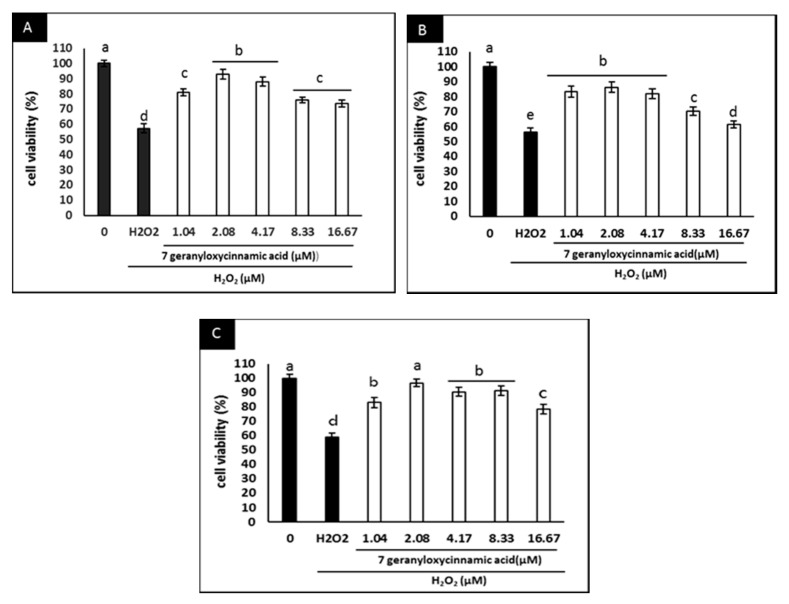
Neuroprotective effect of 7-geranyloxycinnamic acid at various concentrations (1.04–16.67 µM) in differentiated SH-SY5Y cells. The cells were incubated for (**A**) 24 h; (**B**) 48 h; (**C**) 72 h, and exposed to H_2_O_2_ (300 µM) for 4 h. Data are displayed as means ± SD of triplicate experiments; means with different letters denote significant difference (*p* < 0.05).

**Figure 5:**
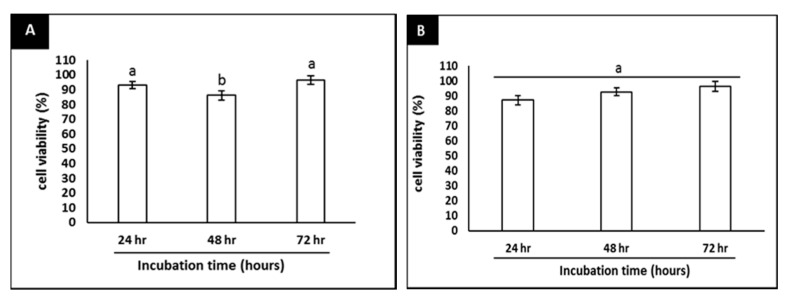
Neuroprotective effect of 7-geranyloxycinnamic acid and curcumin (positive control) in time-dependent manner. (**A**) Represents means of 2.08 µM 7-geranyloxycinnamic acid and exposed to H_2_O_2_ (300 µM) for 4 h. (**B**) Represents means of 5.97 µM curcumin and exposed to H_2_O_2_ (300 µM) for 4 h. Data are displayed as means ± SD of triplicate experiments; means with different letters denote significant difference (*p* < 0.05).

In addition, the neuroprotective effects of 7-geranyloxycinnamic acid in differentiated SH-SY5Y cells and its ability to inhibit the cytotoxicity induced by H_2_O_2_ were investigated. Pretreatment with 7-geranyloxycinnamic acid protected the differentiated cells against H_2_O_2_-mediated cytotoxicity throughout the experimental period (Figure 4A–C). The highest viability was noted when the cells were treated with 2.08 µM 7-geranyloxycinnamic acid, particularly after 72 h of treatment (Figure 5A), which was similar to the treatment of cells with 5.97 µM curcumin plus 4 h exposure to H_2_O_2_ (300 µM) (Figure 5B). Thus, the 7-geranyloxycinnamic acid concentration of 2.08 µM was used in all subsequent studies. 

The *M. lunu-ankenda* plant is widely used as a medicinal plant to treat diabetes mellitus, hypertension, menstrual disorders, fever, and as a tonic [15]. Hydroxycinnamic acids are secondary metabolites isolated from *M. lunu-ankenda* leaves and exhibit antioxidant, anti-inflammatory and neuroprotective activities via impaired H_2_O_2_-mediated oxidative damage [17]. Neuronal cells exposed to H_2_O_2_ displayed enhanced intracellular defense mechanisms, including a rise in endogenous antioxidants [33]. This protection response process, however, fails to protect cells when exposed to excess H_2_O_2_ and prevent cell death [33]. Exogenous antioxidants from plant sources enhance cellular defense systems leading to cells survival [34]. In this study, pre-treatment of the cells with 2.08 µM 7-geranyloxycinnamic acid prevented H_2_O_2_-mediated oxidative damage and showed the highest viability after 72 h of treatment with the phytochemical. In a study performed on SH-SY5Y cells to evaluate the neuroprotective effects of 3,5-di-*O*-caffeoylquinic acid (a cinnamic acid derivative), it was established that the phytochemical exerts its neuroprotective activity by activating ATP production and increasing the expression of PGK1 (phosphoglycerate kinase-1) [35]. Furthermore, pretreatment of these cells with (20 µM) caffeoylquinic impaired apoptosis by inhibiting the activation of caspase-3 and caspase-9 and lowering the Bax/Bcl-2 ratio [36]. Similarly, in another study, the neuroprotective effect of caffeoylquinic acid (CQA) derivatives was established against H_2_O_2_-induced neurotoxicity in SH-SY5Y cells via inhibition of oxidative stress and activation of antioxidant defence [37]. Caffeic acid is a cinnamic acid, its neuroprotective effect at dose 222 µM was demonstrated in the pheochromocytoma (PC12) cell line against H_2_O_2_-induced cytotoxicity [17]. A further study revealed that caffeic acid at concentrations of 55.5 and 111 µM exerts neuroprotective activity against Aβ-induced neurotoxicity in pheochromocytoma (PC12) cells via impaired tau phosphorylation and phosphorylation of GSK-3β (glycogen synthase kinase-3β), impaired oxidative stress and a decrease in intracellular calcium levels [34]. Neuroprotective effects of caffeic acid were also reported in primary mouse cortical neurons against 5-*S*-cysteinyl-dopamine (CysDA)-induced cell injury at dose 1 µM of caffeic acid [19]. Ferulic acid is a hydroxycinnamic acid reported to suppress lipid peroxidation in the synaptosomal system and inhibit free radical damage in cultured neuronal cells at a concentration of 50 µM; in a study conducted in a synaptosome model and in a hippocampal neuronal cell culture, ferulic acid protected against cytotoxicity induced by hydroxyl- and peroxyl radicals [17]. Sinapic acid was studied to evaluate its free radical scavenging ability as exemplified by the 2,2-diphenyl-1-picrylhydrazyl (DPPH) assay. Sinapic acid, at concentration 20 µM, inhibited 33.2% of the DPPH, which is comparable to the scavenging activity of caffeic acid (49.6%) [38]. Cinnamic acids are known as one of the most extensively distributed polyphenols in plants and their neuroprotective effects have been attributed to the presence of the vinylogous CH=CH-COOH group [17]. Polyphenols in general are characterized by low bioavailability as a result of first-pass metabolism, poor cell-membrane permeability and solubility leading to poor intestinal tract permeation [39,40]; more efficient delivery systems are required in order to improve polyphenol bioavailability. Nanoparticle-based delivery systems, including polymeric nanoparticles that encapsulate polyphenolic molecules as nanostructures such as liposomes (LSs) and cyclodextrins (CDs), solid lipid nanoparticles (SLNs), micelles (MCs) and nanospheres (NSs), offer promising strategies to improve polyphenol bioavailability and enhance their therapeutic effects [39,40]. However, cinnamic acid exhibits higher intestinal absorption than other complexed polyphenols, and studies have established that ferulic acid and caffeic acid can penetrate the brain [41]. 

### 2.4. Anti-Apoptotic Effect of 7-Geranyloxycinnamic Acid in SH-SY5Y Cells Monitored Using the AO/PI Double Staining Assay

**Figure 6:**
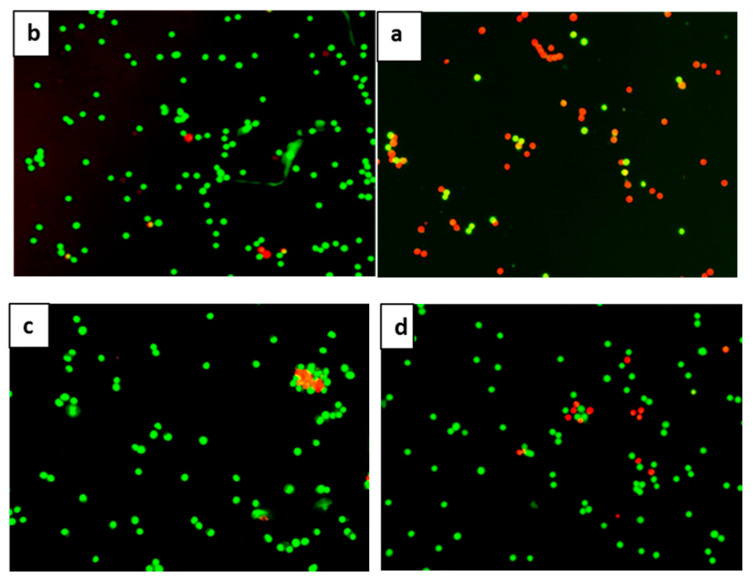

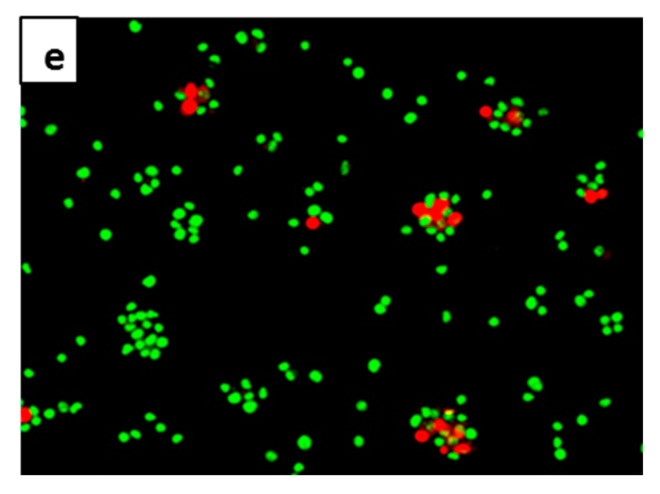
Florescence images of differentiated SH-SY5Y cells stained with acridine orange and propidium iodide (AO/PI) dyes. (**a**) Cells exposed to H_2_O_2_ for 4 h. (**b**) Untreated cells. (**c**) Cells pre-treated for 72 h with 2.08 µM 7-geranyloxycinnamic acid. (**d**) Cells pre-treated for 72 h with 2.08 µM 7-geranyloxycinnamic acid and then exposed to H_2_O_2_ for 4 h. (**e**) Cells pre-treated for 72 h with 5.97 µM curcumin and then exposed to H_2_O_2_ for 4 h. Magnification (×20).

A difference between viable and apoptotic neuron cells was observed by fluorescence microscopy following acridine orange and propidium iodide (AO/PI) staining. Viable cells were characterized by green stained nuclei, whereas cells exposed to H_2_O_2_ (apoptotic cells) stained red (Figure 6a–e). More than half of the cells stained red 4 h after treatment with H_2_O_2_ (Figure 6a). In cells pretreated with 7-geranyloxycinnamic acid prior to H_2_O_2_ exposure, a high percentage of the cell nuclei were stained green (Figure 6d). This picture is similar to what was observed when the cells were treated with curcumin prior to H_2_O_2_ (Figure 6e). Untreated cells (Figure 6b) and 72 h treated cells with 2.08 µM 7geranyloxycinnamic acid (Figure 6c) displayed a high percentage of green stained nuclei commensurate with normal viable cell appearance. It has already been demonstrated that the nucleus of viable cells stains green with the AO dye that permeates the cellular membrane, but stains red with the PI dye only when the cellular membrane has been disrupted [42,43,44]. In the present study, 7-geranyloxycinnamic acid clearly afforded protection against H_2_O_2_-induced cytotoxicity since cells mostly stained green. Polyphenols are well known protective agents of mitochondrial integrity via modulating apoptosis and antioxidant enzymes [17]. Studies have shown that polyphenols exert their neuroprotective activity by upregulating anti-apoptotic Bcl-2 (B-cell lymphoma 2), superoxide dismutase (SOD) and glutathione peroxidase (GPX1) [45,46,47], and downregulating c-Jun, JNK, interferon-γ inducible protein and COX-2 [44]. Similarly, ferulic acid (another cinnamic acid derivative) suppressed mitochondrial apoptosis by upregulating Bcl-2, heme oxygenase 1 (HO-1) and SOD, but downregulating Bad/Bax (BCL2-associated agonist of cell death/BCL2-associated X protein) expression and COX-2 activity [48,49,50]. Furthermore, ferulic acid exerts its anti-apoptotic effect by downregulating the p38 MAPK pathway, protecting cell membrane, decreasing production of PGE2, and by lowering intracellular free Ca^2+^ ion and MDA levels in PC12 cells [41]. In a study undertaken to evaluate the effects of a group of bioavailable polyphenols against H_2_O_2_-induced cell death in the SH-SY5Y cell line, gallic acid (phenolic acid) impaired apoptosis of neuronal cells through decreasing ROS levels and preventing the activation of caspase-3 through the mitochondrial apoptotic pathway [51].

### 2.5. Confirmation of the Anti-Apoptotic Effect of 7-Geranyloxycinnamic Acid in Differentiated SH-SY5Y Cells Using the Annexin V-FITC Assay

**Figure 7:**
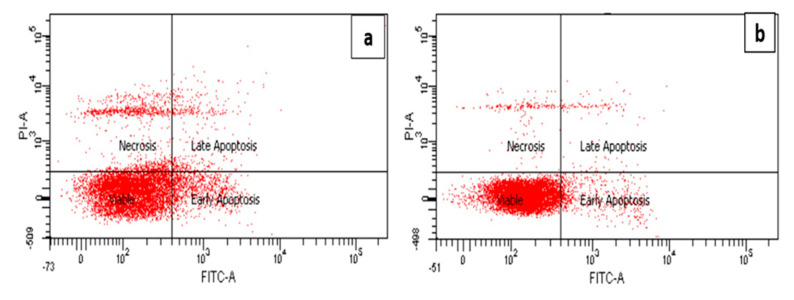

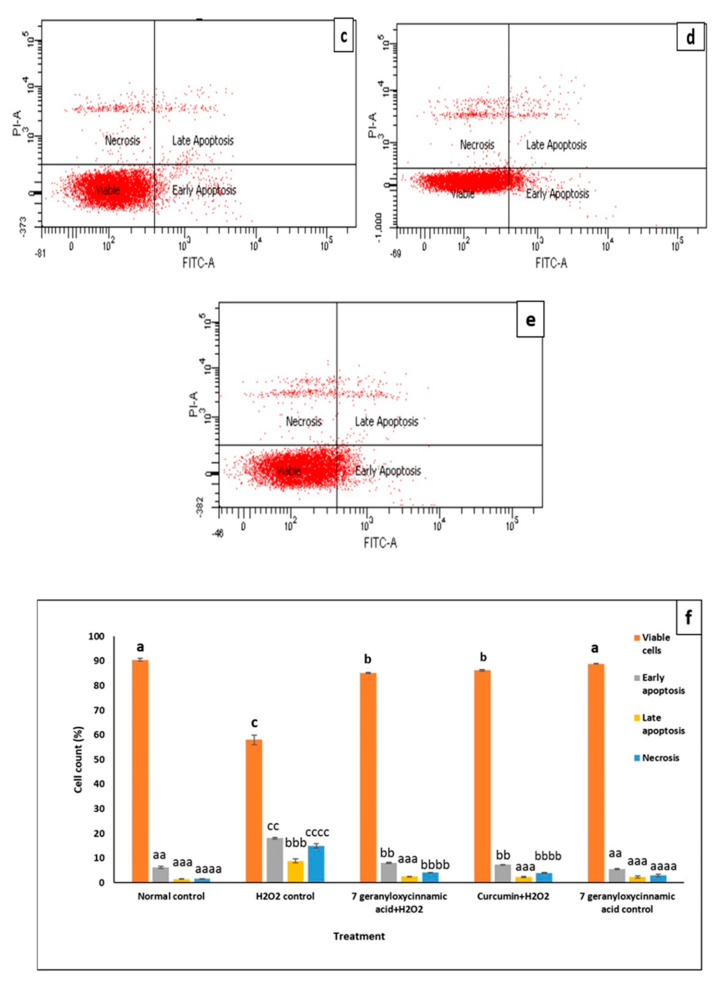
Annexin V-FITC assay of differentiated SH-SY5Y cells using flow cytometry. (**a**) Cells exposed to H_2_O_2_ for 4 h. (**b**) Untreated cells. (**c**) Cells pre-treated for 72 h with 2.08 µM 7-geranyloxycinnamic acid. (**d**) Cells pre-treated for 72 h with 2.08 µM 7-geranyloxycinnamic acid and then exposed to H_2_O_2_ for 4 h. (**e**) Cells pre-treated for 72 h with 5.97 µM curcumin and then exposed to H_2_O_2_ for 4 h. Data are displayed as means ± SD of triplicate experiments; means with different letters denote significant difference (*p* < 0.05) (**f**).

The Annexin V-FITC assay employing flow cytometry was performed in order to evaluate the distribution of the cells at the four different quadrants, namely viable cells, early apoptosis, late apoptosis and necrosis. Healthy cells appeared in the left-lower quadrant (Annexin-V−/PI−), the early apoptotic cells in the right-lower quadrant (Annexin-V+/PI−), whereas in the right-upper quadrant (annexin-V+/PI+), late apoptotic cells were present and, finally, necrotic cells appeared at the left-upper quadrant (annexin-V−/PI+) as shown in Figure 7. The neuroprotective activity of 7-geranyloxycinnamic acid against H_2_O_2-_induced is evidenced by the much lower number of apoptotic cells and increased cell viability in the 7-geranyloxycinnamic acid pre-treated cells prior to H_2_O_2_ exposure (Figure 7d) in comparison with cells treated only with H_2_O_2_ (Figure 7a). Clearly, the cells pretreated with either 7-geranyloxycinnamic acid or curcumin, the positive control, showed a lower level of apoptosis and high cell viability, and appeared similar to the normal control (untreated) cells (Figure 7f). This may be due to the ability of 7-geranyloxycinnamic acid in protecting lipid asymmetry membrane and preventing phosphatidylserine (PS) translocation to cytoplasm, and maintaining the distribution of phosphatidylserine (PS) in the cytoplasmic leaflet [52]. Phosphatidylserine (PS) translocation, due to disruption of the plasma membrane asymmetry, may occur as a result of cell exposure to oxidative stress injury triggering apoptosis [53]. 

### 2.6. Surface Ultrastructural Preservation of SH-SY5Y Cells Pre-Treated with 7-Geranyloxycinnamic Acid

**Figure 8:**
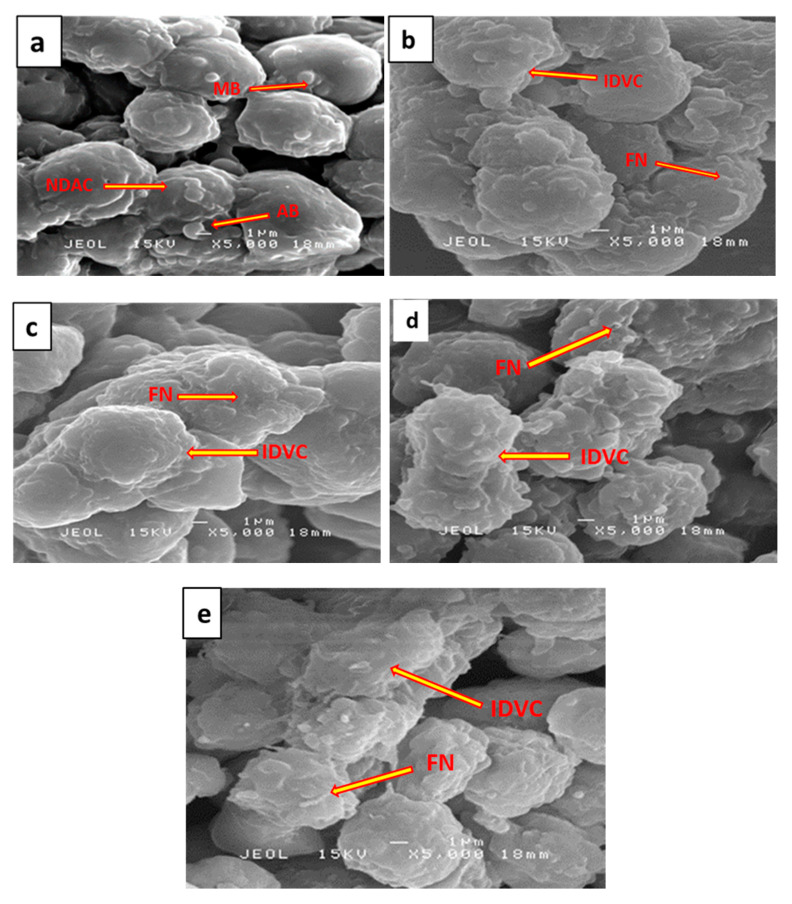
Surface ultrastructural assessment of differentiated SH-SY5Y cells by SEM. (**a**) Cells exposed to H_2_O_2_ for 4 h. (**b**) Untreated cells. (**c**) Cells pretreated for 72 h with 2.08 µM 7-geranyloxycinnamic acid. (**d**) Cells pre-treated for 72 h with 2.08 µM 7-geranyloxycinnamic acid and then exposed to H_2_O_2_ for 4 h. (**e**) Cells pre-treated for 72 h with 5.97 µM curcumin and then exposed to H_2_O_2_ for 4 h. AB = apoptotic bodies, FN = folded neurites, NDAC = neurite disrupted apoptotic cells, IDVC = intact differentiated viable cells, MB = membrane blebbing. Magnification (× 5000).

The surface morphological assessment of differentiated SH-SY5Y cells, performed by using scanning electron microscopy (SEM) (Figure 8a–e), revealed interesting changes in the cells exposed to H_2_O_2_ only (Figure 8a), and in the cells pre-treated with 7-geranyloxycinnamic acid followed by exposure to H_2_O_2_ (Figure 8d). Morphological abnormalities, including membrane blebbing, cell shrinkage and neurite disruption [54], were observed in all H_2_O_2_-exposed cells (Figure 8a). Cells with 7-geranyloxycinnamic acid prior to H_2_O_2_ (Figure 8d) showed intact surface features similar to the curcumin-pre-treated group (positive control) (Figure 8e) with integrated cytosol and folded neurites. Membrane integrity was observed in untreated cells (Figure 8b) and in cells pre-treated with 7-geranyloxycinnamic acid only (Figure 8c). These findings indicate the potential of 7-geranyloxycinnamic acid to preserve surface structure and enhance cell viability.

### 2.7. Ultrastructural Protection of Differentiated SH-SY5Y Cells by 7-Geranyloxycinnamic Acid Pre-Treatment as Determined by TEM

**Figure 9:**
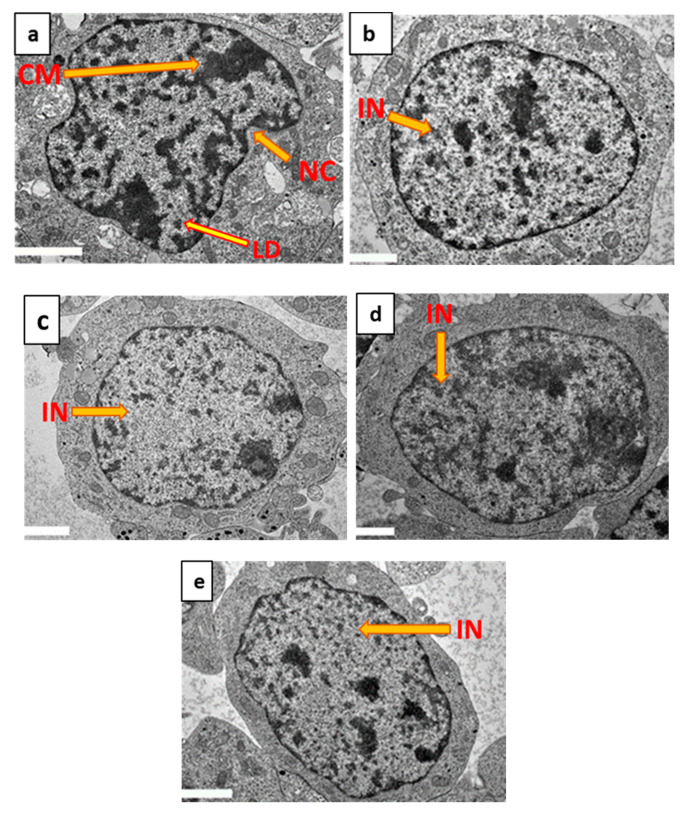
Ultrastructural assessment of differentiated SH-SY5Y cells by TEM. (**a**) Cells exposed to H_2_O_2_ for 4 h. (**b**) Untreated cells. (**c**) Cells pretreated for 72 h with 2.08 µM 7-geranyloxycinnamic acid. (**d**) Cells pre-treated for 72 h with 2.08 µM 7-geranyloxycinnamic acid and then exposed to H_2_O_2_ for 4 h. (**e**) Cells pre-treated for 72 h with 5.97 µM curcumin and then exposed to H_2_O_2_ for 4 h. IN = intact nucleus, CM = chromatin margination, NC = nuclei convolution, LD = lipid droplet. Magnification (×3000).

Examination of the ultrastructure of differentiated SH-SY5Y cells using TEM (Figure 9a–e) revealed abnormalities following exposure to H_2_O_2_ only. These abnormal morphological features include the apoptotic characteristics of nuclear convolution, nuclear shrinkage, chromatin margination and chromatin condensation [55,56] (Figure 9a). In differentiated cells pre-treated with either 7-geranyloxycinnamic acid (Figure 9d) or curcumin (Figure 9e) plus H_2_O_2_, these abnormal features were absent and cells resembled those receiving no treatment (Figure 9b) and 7-geranyloxycinnamic acid-treated cells (Figure 9c). As 7-geranyloxycinnamic acid prevents the appearance of typical apoptotic characteristics, it may be inferred that 7-geranyloxycinnamic acid exhibits potential neuroprotective activity, presumably by inhibiting oxidative stress.

## 3. Materials and Methods 

### 3.1. Compound

7-geranyloxycinnamic acid was kindly supplied by the Institute of Bioscience, Universiti Putra Malaysia, UPM Serdang, Selangor, Malaysia. The isolated compound was characterized using nuclear magnetic resonance (NMR) and direct injection probe (DIP) techniques (data not shown).

### 3.2. Cell Culture and Maintaining

Human neuroblastoma SH-SY5Y cells were obtained from the Institute of Bioscience (IBS), Universiti Putra Malaysia (UPM). SH-SY5Y cells are established as an NDDs model and could be differentiated into terminal neurons by retinoic acid (RA)-treatment. The cells were maintained in a 1:1 mixture of Dulbecco Modified Eagle Medium and Ham’s F12 (DMEM/Hams’ F12) (Nacalai, Kyoto, Japan) supplemented with 10% fetal bovine serum (FBS), 2 mM of L-glutamine and 1% penicillin/streptomycin (Nacalai, Kyoto, Japan) in a 5% CO_2_ incubator at 37 °C. Cell culture media were replaced every 2 days, and, at 80–90% of confluence, the cells were sub-cultured [25].

### 3.3. Cell Differentiation 

SH-SY5Y cells were seeded (1 × 10^5^ cells/well) in 6-well plates. The cells were incubated for 24 h at 37 °C in a 5% CO_2_ incubator, and then differentiated by addition of 10 µM all *trans* retinoic acid (RA), dissolved in DMEM/F12 media supplemented with 3% heat-inactivated FBS, and further incubated in the dark at 37 °C in a 5% CO_2_ incubator for 7 days. The RA-treated media were replaced every day for 7 days, and then visualized under a fluorescence microscope (phase contrast, Zeiss Axio Vert A1, Jena, Germany) to monitor the differentiation and to measure the neurite length of undifferentiated and differentiated SH-SY5Y cells [25]. Finally, differentiation of SH-SY5Y cells by all *trans* retinoic acid was confirmed by immunocytochemistry, where NeuN expression was detected by an FITC-conjugated secondary anti-rabbit antibody (Alexa fluorophore-488 secondary antibody conjugate). 

### 3.4. Immunocytochemical (ICC) Analysis

SH-SY5Y cells differentiation by 10 µM all *trans* retinoic acid (RA) was confirmed using the ICC assay according to the kit protocol: the cells were seeded at a density of 2 × 10^4^ cells/well in 24-well plates and differentiated by RA as previously described. The differentiated cells were washed 3 times with cold PBS (0.01M phosphate buffer, 0.0027 M potassium chloride and 0.137 M sodium chloride) pH 7.4, at 25 °C, were then fixed for 30 min at 25 °C by addition of 300 µL fixation solution (4% paraformaldehyde; PFA, 1M NaOH and PBS). The cells were subsequently incubated for 15 min at 25 °C with 300 µl permeation solution (1% Triton X-100 and 99% PBS). After washing once again with PBS, 300 µl blocking solution (10% goat serum, 10% tween 20, 0.3% bovine serum albumin and PBS) was added to the cells for 30 min at 25 °C. After washing three times with PBS, the cells were incubated overnight at 4 °C with a cytoplasmic neuron-specific protein (antibody for Class III β-tubulin (Tuj-1)) and blocking solution in a ratio of 1:200. On the following day, the cells were washed three times with PBS and incubated with secondary antibody conjugate (Alexa fluorophore-488) and blocking solution in a ratio of 1:200 in the dark at 25 °C for 2 h, and then incubated for a further 10 min with 4′,6-Diamidine-2′-phenylindole dihydrochloride (DAPI) dye [25]. Cells were visualized under an inverted light fluorescence microscope (Zeiss Axio Vert A1, Jena, Germany).

### 3.5. Neuroprotection and Cell Viability Assay

The 3-(4,5-dimethylthiazol-2-yl)-2,5-diphenyltetrazolium bromide (MTT) assay was conducted to assess the effect of 7-geranyloxycinnamic acid on cell viability and its neuroprotective effect against H_2_O_2_-induced oxidative damage as described by Jaafaru et al. [25]. The cells (1 × 10^4^) were seeded in 96-well plates to which medium was added (100 µL per well), and then differentiated for 7 days as described above. SH-SY5Y cells were pre-treated with serially diluted concentrations of 7-geranyloxycinnamic acid (1.04–16.67 µM) [26] in a time-dependent manner (24, 48 and 72 h) to evaluate its effect on cell viability. The cells were incubated with the 20 µL of the MTT solution (5 mg/mL) for 4 h in the dark, and then 100 µL of dimethyl sulfoxide (DMSO) to solubilize the formed formazan. Absorbance was measured at 570 nm using a microplate reader (Synergy H1, BioTek, Winooski, VT, USA). The MTT assay was also used to confirm the neuroprotective effect of 7-geranyloxycinnamic acid, where the differentiated cells were incubated with 7-geranyloxycinnamic acid (serially diluted, 1.04–16.67 µM) for 24, 48 and 72 h prior to the addition of H_2_O_2_ (300 µM) [25] for 4 h.

### 3.6. Acridine Orange (AO) and Propidium Iodide (PI) Staining Assay

Approximately 1 × 10^5^ cells/well of SH-SY5Y were seeded into a 6-well plate and differentiated as described above for 7 days. After pretreatment of the cells for 72 h with 7-geranyloxycinnamic acid followed by a 4 h exposure to H_2_O_2_ (300 µM) [25], the cells were trypsinized by addition of 500 µL trypsin and then incubated for 4 min at 37 °C in a 5% CO_2_ incubator, neutralized, centrifuged, washed twice and re-suspended in PBS. A ten microliter aliquot of the cell suspension was mixed with 1 µL AO (10 mg/mL) and 10 µL PI (1 mg/mL) and incubated for 15 min in the dark at 25 °C and was then placed on a glass slide. The cells were viewed using an inverted fluorescence microscope (Zeiss Axio Vert A1, Jena, Germany) [25,27].

### 3.7. Flow Cytometry Analysis

The Annexin V-FITC staining assay kit (BD Pharmingen, Tokyo, Japan) was used to detect cell apoptosis. The assay was carried out according to the kit protocol; in brief, approximately 1 × 10^5^ of SH-SYSY cells/well were seeded into 6-well plates and differentiated for 7 days. The cells were pre-treated with 7-geranyloxycinnamic acid for 72 h followed by 4 h exposure to H_2_O_2_. The cells were trypsinized by addition of 500 µL trypsin, incubated for 4 min at 37 °C in a 5% CO_2_ incubator, neutralized, centrifuged and washed with PBS, and were finally resuspended in (1X) binding buffer. Five microliters of annexin V-FITC solution and 5 µL of propidium iodide (PI) were mixed with the cell suspension (40 µL) and incubated in the dark for 15 min at 25 °C. Subsequently, 450 µL 1X binding buffer was added, and, after vortexing and filtration, the samples were analyzed using a flow cytometer [25].

### 3.8. Scanning Electron Microscopy (SEM)

Approximately 1 × 10^6^ SH-SY5Y cells/ flask were seeded in T25 mL flasks. The cells were differentiated and treated with 7-geranyloxycinnamic acid for 72 h followed by a 4-h exposure to H_2_O_2_. The pre-treated cells were washed twice with PBS after trypsinization. The treated cells were fixed for 6 h with 4% glutaraldehyde and for 2 h with 1% osmium tetroxide. After washing 3 times with 0.1 M sodium cacodylate buffer, each for 10 min, the cells were dehydrated using 35, 50, 75 and 95% acetone. Further dehydration was performed 3 times using 100% acetone, and the samples were dried for 30 min on a critical dryer. Coating of the dried pellet with gold particles was performed directly after mounting, and the cells were examined under a scanning electron microscope (JSM 6400, Joel, San Francisco, CA, USA) [25].

### 3.9. Transmission Electron Microscopy (TEM)

SH-SY5Y cells were seeded in T25 mL flasks (1 × 10^6^ cells/flask), differentiated as previously described and treated with 7-geranyloxycinnamic acid for 72 h followed by H_2_O_2_ for four hours. The cells were subsequently trypsinized and washed twice with PBS. Cells were fixed using 4% glutaraldehyde for 6 h and 1% osmium tetroxide for 2 h, and thereafter dehydrated using various acetone concentrations as described above. Using a mixture of acetone and resin, the cells were infiltrated in three steps, for 60 min, 120 min and overnight, in a ratio 1:1, 1:3 and 100% resin, respectively. The infiltrated cells were embedded by insertion into a beam capsule filled with resin. Following polymerization by incubating in an oven at 60 °C for 2 days, the specimen was then cut in to sections of 1 µm thickness using an ultramicrotome. The sections were stained with toluidine, and the section thickness was reduced to 60–90 nm. The specimen was stained with 4% uranyl acetate for 15 min and with 1% lead citrate for 10 min to scatter imaging electrons and provide contrast between different structures of the sample. The samples were viewed using a transmission electron microscope (JEM-2100F, Joel, San Francisco, CA, USA) [25].

### 3.10. Statistical Analysis

The data are presented as means ± standard deviation. One-way analysis of variance (ANOVA) with Tukey’s multiple comparison was conducted to determine differences between the means using the Minitab Statistical Package, version 18 (Minitab Inc. State College, PA, USA) with significant difference considered at *p <* 0.05.

## 4. Conclusions

It was demonstrated in the present studies that 7-geranyloxycinnamic acid protects neuronal cells against H_2_O_2_-mediated cell death. 7-geranyloxycinnamic acid appears to act by maintaining membrane integrity, and internal features where it prevents the occurrence of nuclear shrinkage, nuclear convolution, chromatin condensation and chromatin margination of differentiated neuronal cells. The protective effect of 7-geranyloxycinnamic acid may be also related to its ability to impair apoptosis. The current findings raise the possibility that 7-geranyloxycinnamic acid may prove beneficial in the prevention and treatment of neurodegenerative diseases.
